# Relationship between psychosocial risk factors at work and musculoskeletal symptoms in university professors

**DOI:** 10.47626/1679-4435-2025-1413

**Published:** 2025-08-25

**Authors:** Carlos Manoel Lopes Rodrigues

**Affiliations:** 1 School of Health Sciences, Centro Universitário de Brasília, Brasília, DF, Brazil; 2 Postgraduate Program in Clinical Psychology and Culture, Universidade de Brasília, Brasília, DF, Brazil

**Keywords:** cost of illness, cumulative trauma disorders, occupational health, faculty., efeitos psicossociais da doença, distúrbios osteomusculares relacionados ao trabalho, saúde ocupacional, docentes.

## Abstract

**Introduction:**

Musculoskeletal disorders are prevalent among university professors. With the expansion of private higher education and the increasing demands on academic staff, psychosocial risk factors may exacerbate these conditions beyond ergonomic challenges.

**Objectives:**

To investigate the relationship between psychosocial risk factors and musculoskeletal symptoms among university professors in the private sector.

**Methods:**

This quantitative, cross-sectional, and correlational study involved 122 university professors. Data were collected using the Nordic Musculoskeletal Questionnaire and the Scale for Evaluating Psychosocial Stressors in the Workplace. Analyses included descriptive statistics, point-biserial correlations, and Structural Equation Modeling (SEM) to assess latent relationships between psychosocial factors and musculoskeletal outcomes.

**Results:**

The structural model demonstrated an adequate fit to the data (χ^2^_[38]_ = 58.590; p = 0.05124; comparative fit index = 0.98; Tucker-Lewis index = 0.97; standardized root mean square residual = 0.08; root mean square error of approximation = 0.06 [95%CI 0.02-0.06]), confirming a significant association between psychosocial factors and musculoskeletal symptoms. Psychosocial risk factors contributed to the occurrence of musculoskeletal symptoms in the past 12 months (β = 0.40; p < 0.001), work impairments (β = 0.34; p = 0.001), recent symptoms (β = 0.32; p < 0.001), and health care-seeking behavior (β = 0.52; p < 0.001). The most influential factors were job insecurity, work-family conflict, and role overload.

**Conclusions:**

Psychosocial factors in academic work significantly impact the manifestation of musculoskeletal symptoms and their functional consequences. Managing these factors is essential for preventing and mitigating their effects on faculty health.

## Abstract (pt)

**Introdução:**

Disfunções osteomusculares são frequentes no exercício da docência. Ante a expansão do ensino superior privado e o consequente aumento das exigências sobre os docentes, além das exigências ergonômicas, fatores de risco psicossociais podem agravar esses problemas.

**Objetivos:**

Investigar a relação entre fatores de risco psicossociais e sintomas osteomusculares em professores universitários do setor privado.

**Métodos:**

Estudo quantitativo, transversal e correlacional, com 122 professores universitários. Foram utilizados o Nordic Musculoskeletal Questionnaire e a Escala de Avaliação de Estressores Psicossociais no Contexto Laboral. A análise de dados incluiu estatísticas descritivas, correlação ponto-bisserial e modelagem de equações estruturais para avaliar as relações latentes entre os fatores psicossociais e os desfechos osteomusculares.

**Resultados:**

O modelo estrutural apresentou um ajuste adequado aos dados (χ^2^_(38)_ = 58,590; p = 0,05124; *comparative fit index* = 0,98; índice Tucker-Lewis = 0,97; *standardized root mean square residual* = 0,08; *root mean square error of approximation* = 0,06 [intervalo de confiança de 95% 0,02-0,06]), confirmando a relação significativa entre fatores psicossociais e sintomas osteomusculares. Os resultados indicaram influência dos fatores de risco psicossociais na ocorrência de sintomas osteomusculares nos últimos 12 meses (β = 0,40; p < 0,001), impedimentos no trabalho (β = 0,34; p = 0,001), sintomas recentes (β = 0,32; p < 0,001) e busca por tratamento (β = 0,52; p < 0,001). Os fatores mais impactantes foram insegurança na carreira, conflito trabalho-família e sobrecarga de papéis.

**Conclusões:**

Os fatores psicossociais no trabalho docente exercem um impacto significativo na manifestação de sintomas osteomusculares e seus desdobramentos funcionais. Gerir esses fatores é essencial para prevenir e mitigar os impactos na saúde dos docentes.

## INTRODUCTION

In recent years, higher education in Brazil has undergone significant expansion, with a substantial increase in the number of higher education institutions (HEIs). The broadening of access was driven by public policies for student financing, such as the Student Financing Fund (Fundo de Financiamento Estudantil, FIES) and the University for All Program (Programa Universidade para Todos, Prouni), in addition to regulatory flexibility that allowed for the emergence of new institutions and teaching modalities, such as distance learning.^1^ The growth of the private sector has been particularly pronounced, accounting for most enrollments in Brazilian higher education (2,264 private HEIs).^2^

This scenario has intensified competition among institutions, which now compete for students through differentiators such as infrastructure, pedagogical innovation, scholarship offerings, and marketing strategies. The entry of large educational groups into the market has reinforced a business-oriented approach in the sector, leading to restructuring efforts and acquisitions of smaller colleges.

In this setting, academic work in higher education has become even more demanding - not only due to the traditional responsibilities of the profession, which include planning, creativity, and conflict mediation, but also because of the pressures resulting from the commercialization of education and the increasing competition among HEIs.^3^ Academic staff working conditions frequently involve long working hours, ergonomic issues, inadequate postures, and repetitive activities such as extensive typing and the preparation of educational materials.^3^,^4^ These physical factors can contribute to the development of musculoskeletal disorders, including muscle pain, repetitive strain injuries (RSI), and other musculoskeletal conditions.^5^,^6^

However, musculoskeletal disorders are not solely the result of physical work demands but are also influenced by contextual factors.^7^-^9^ Problems related to work organization and management can exacerbate or trigger physical symptoms.^10^-^12^ These factors, classified as psychosocial factors, play a significant role in this process, influencing both the likelihood and severity of musculoskeletal problems.^7^,^8^,^13^

Psychosocial risks at work refer to potential negative health and well-being outcomes resulting from exposure to adverse working conditions.^14^ These risks manifest as tangible consequences, such as chronic stress, mental health disorders, professional burnout, and musculoskeletal dysfunctions.^15^ Psychosocial risk factors (PRFs), on the other hand, are the conditions or characteristics of the work environment that contribute to these outcomes, such as excessive workload, low autonomy, precarious employment relationships, interpersonal conflicts, and exposure to physical and psychological violence.^14^

The recognition of the importance of psychosocial risks at work has grown globally, driven by scientific evidence demonstrating their direct relationship with the mental and physical health of workers.^16^ In Brazil, this recognition was solidified with the update of Regulatory Standard-1 (Norma Regulamentadora-1, NR-1), which now requires the identification and management of psychosocial risks as part of the Occupational Health Management framework.

The inclusion of these risks in Brazilian regulations represents a significant shift in how occupational health is understood, extending beyond the traditional focus on physical and chemical hazards. With the new NR-1, employers are responsible for assessing and implementing preventive measures to minimize the impacts of stress, excessive workload, lack of autonomy, and other psychosocial factors affecting the health of workers. This change marks a major advancement in national labor regulations, aligning Brazil with international best practices and reinforcing the importance of healthy and sustainable work environments.^17^

Effective actions to mitigate the risk of musculoskeletal disorders in the workplace must necessarily include an investigation of associated PRFs. Thus, this study aimed to identify the occurrence of musculoskeletal symptoms and their relationship with exposure to PRFs at work among university professors in private HEIs. Understanding this relationship can contribute to the development of prevention strategies and the mitigation of the impacts of working conditions on faculty staff health, fostering healthier and more sustainable academic environments.

## METHODS

This study adopted a quantitative, cross-sectional, and correlational approach.

### SAMPLE

The study included 122 university professors from a private HEI. The sample comprised 67 women (54.92%) and 55 men (45.08%), aged between 27 and 64 years (mean [M] = 40.11; SD = 6.99), with teaching experience ranging from 2 to 35 years (M = 7.85, SD = 9.80). The weekly workload varied from 16 to 40 teaching hours (M = 25.50; SD = 8.50).

As an inclusion criterion, participants were required to have at least 1 year of teaching experience in higher education and to have teaching as their primary professional activity at the time of data collection.

### INSTRUMENTS

#### Sociodemographic questionnaire

This questionnaire was used to collect data on sex, marital status, educational level, age, weekly workload, and years of teaching experience in higher education.

#### Scale for Assessing Psychosocial Stressors in the Workplace (Escala de Avaliação de Estressores Psicossociais no Contexto Laboral, EAEPCL)^18^

The EAEPCL was developed to measure the impact of stress factors on professional activities. The scale consists of 35 items distributed across seven factors: role conflict and ambiguity, role overload, lack of social support, job insecurity, lack of autonomy, work-family conflict, and responsibility pressure.

#### Brazilian Version of the Nordic Musculoskeletal Questionnaire (NMQ)^19^

The NMQ is used to identify the occurrence of musculoskeletal symptoms in different areas of the body over the last 12 months and the last 7 days, as well as disability and whether medical care was sought due to symptoms based on a dichotomous yes/no scale. The instrument is widely used in epidemiological and ergonomic studies to assess the occurrence of musculoskeletal symptoms in different professional contexts.^20^,^21^

### PROCEDURES

The instruments were administered online, with dissemination through the internal network of the HEI. The institution provided formal authorization, and the researcher had no professional ties to it to avoid potential conflicts of interest.

The questionnaires were sent via an electronic form, accompanied by the Informed Consent Form. Responses were coded to ensure anonymity and data confidentiality.

This study is part of a broader project on faculty staff health and was approved by the Ethics Committee of the researcher’s institution (approval number 1218747; CAEE number 46687815.5.0000.0023).

### DATA ANALYSIS

The collected data were analyzed using the R statistical software. Descriptive and inferential statistics were used to examine variable distributions and identify potential patterns.

Initially, normality tests and multivariate outlier detection were performed. The psychometric properties of the instruments used in this study were assessed through internal consistency using the following coefficients: Cronbach’s alpha (α) and Guttman’s lambda 2 (λ_2_) for the EAEPCL, and Kuder-Richardson formula (KR-20) for the NMQ.

The relationships between variables were examined using the point-biserial correlation coefficient (r_pb_), which is appropriate for assessing associations between continuous and dichotomous variables. Furthermore, relationships between latent variables and clinical outcomes were analyzed through structural equation modeling (SEM), using a robust estimator (weighted least squares mean and variance adjusted), which is suitable for handling non-normally distributed data derived from psychometric scales.^22^,^23^ Model fit was assessed using chi-square (χ^2^) indices, for which non-significant results (p > 0.05) are expected.^24^ Additionally, the comparative fit index (CFI) and Tucker-Lewis index (TLI) were considered acceptable when above 0.90.^25^ The root mean square error of approximation (RMSEA) and standardized root mean square residual (SRMR) were also evaluated, with values below 0.08 considered adequate.^26^,^27^

## RESULTS

Normality tests indicated a violation of the normality assumption. However, this did not affect the analyses, as the methods used (r_pb_ and SEM) do not require normality assumptions. Regarding the psychometric properties of the instruments, adequate reliability coefficients were identified for the EAEPCL (α = 0.94, λ_2_ = 0.95) and the NMQ (KR-20 = 0.87, p < 0.001, 95%CI: 0.79-0.91).

The frequency analysis of musculoskeletal symptoms revealed that most participants (68.03%) reported at least one complaint in the last 12 months, with an average of 5.91 different complaints per participant (SD = 1.26). Regarding work-related impairments, participants reported an average of 4.60 work absences due to symptoms in the last year (SD = 0.79) and an average of 4.86 visits to specialized health care providers (SD = 1.09). For musculoskeletal complaints during the week of data collection, participants reported an average of 5.03 affected regions (SD = 0.99).

Considering the affected body regions, the back (upper and lower) was the most commonly affected area in the last 12 months, followed by the neck and shoulders (Table 1). The prevalence of symptoms in the last 12 months was highest in the upper back (68.03%), followed by the neck (61.48%), lower back (56.56%), and shoulders (52.46%). The least frequently reported symptoms during this period were in the hip/thigh region (23.77%). Regarding functional impairments caused by musculoskeletal symptoms in the last 12 months, the highest rates were observed in the upper back (23.77%), neck (22.95%), and lower back (16.39%), while the lowest rate was recorded for the hip/thigh region (6.56%).

**Table 1:** Absolute and relative frequencies of musculoskeletal symptoms

	Symptoms 12 months		Impairments 12 months		Medical visits 12 months		Symptoms 7 days	
	n	%	n	%	n	%	n	%
Neck	75	61.48	28	22.95	23	18.85	45	36.89
Shoulders	64	52.46	21	17.21	22	18.03	45	36.89
Back (upper)	83	68.03	29	23.77	28	22.95	52	42.62
Wrist/hands	50	40.98	14	11.48	22	18.03	28	22.95
Back (lower)	69	56.56	20	16.39	35	28.69	31	25.41
Hip/thighs	29	23.77	8	6.56	25	20.49	13	10.66
Knees	58	47.54	17	13.93	34	27.87	22	18.03
Ankle/feet	58	47.54	11	9.02	23	18.85	16	13.11

Regarding medical visits in the last 12 months, participants most frequently saw doctors for symptoms in the lower back (28.69%), knees (27.87%), and upper back (22.95%). The lowest rates of medical visits were reported for symptoms in the wrists/hands (18.03%) and ankles/feet (18.85%). In the last 7 days, the highest occurrence of symptoms was reported in the upper back (42.62%), followed by the neck and shoulders (36.89%). The lowest frequencies of recent symptoms were observed in the hip/thigh region (10.66%) and the ankles/feet (13.11%).

Regarding potential differences between men and women in musculoskeletal issues, no statistically significant differences were found between the groups for any of the analyzed variables.

The analysis of r_pb_ between PRFs at work and musculoskeletal symptoms revealed significant associations (Table 2). Job insecurity, work-family conflict, and role overload were most consistently associated with the presence of musculoskeletal symptoms, impairments, and medical visits. Lack of social support was the factor most strongly associated with the need for medical care (r_pb_ = 0.53, p < 0.01), while role conflict and ambiguity, lack of autonomy, and responsibility pressure also showed significant, though less pronounced, associations.

**Table 2:** Correlations between exposure to PRFs at work and musculoskeletal symptoms

	Musculoskeletal symptoms							
	Symptoms 12 months		Impairments 12 months		Medical care 12 months		Symptoms 7 days	
	r_pb_	95%CI	r_pb_	95%CI	r_pb_	95%CI	r_pb_	95%CI
RAC	0.26^*^	0.06-0.45	0.03	-0.13-0.18	0.37^*^	0.22-0.51	0.13	-0.05-0.29
RO	0.28^*^	0.11-0.44	0.37^*^	0.24-0.49	0.33^*^	0.18-0.46	0.27^*^	0.07-0.47
LSS	0.22^**^	0.04-0.38	0.39^*^	0.17-0.55	0.53^*^	0.33-0.67	0.00	-0.17-0.17
JI	0.37^*^	0.21-0.52	0.30^*^	0.13-0.45	0.42^*^	0.28-0.54	0.37^*^	0.23-0.49
LA	0.25^*^	0.06-0.43	0.31^*^	0.15-0.45	0.47^*^	0.31-0.61	0.09	-0.14-0.27
WFC	0.35^*^	0.21-0.48	0.27^*^	0.13-0.38	0.37^*^	0.26-0.47	0.38^*^	0.24-0.51
RAP	0.22^**^	0.05-0.39	0.05	-0.12-0.22	0.27^*^	0.15-0.40	0.22^**^	0.05-0.37

RAC = role ambiguity and conflict; WFC = work-family conflict; LA = lack of autonomy; PRFs = psychosocial risk factors; LSS = lack of social support; JI = job insecurity; RAP = responsibility-associated pressure; r_pb_ = point-biserial correlation coefficient; RO = role overload.

* = p < 0.01;

** = p < 0.05.

**Figure 1:**
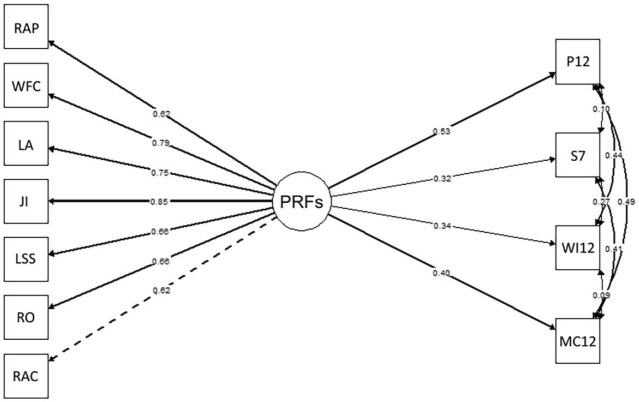
Structural model. P12 = problems in the last 12 months (work impairment in the last 12 months [WI12]; need for medical care in the last 12 months [MC12]; symptoms in the last 7 days [S7]); RAC = role ambiguity and conflict; WFC = work-family conflict; LA = lack of autonomy; PRFs = psychosocial risk factors; LSS = lack of social support; JI = job insecurity; RAP = responsibility-associated pressure; RO = role overload.

Based on these results, SEM was employed to test the structural relationship between psychosocial factors and musculoskeletal outcomes, assessing the overall impact of PRFs on health-related variables (Figure 1).

The evaluation of the proposed structural model showed acceptable fit indices. The chi-square test yielded significant values (χ^2^_(38)_ = 58.590, p = 0.05124), suggesting that the model fits the data adequately. However, since this statistic test is sensitive to sample size, its results should be interpreted alongside that of other fit indices, as the significance level is very close to the threshold (p < 0.05).

The CFI (0.98) and TLI (0.97) indicated a very good fit, as both values exceed the recommended criterion of 0.95. The RMSEA (0.06 [95%CI: 0.02-0.06]) suggested an acceptable fit, given that values below 0.08 indicate an adequate fit, while values below 0.05 are considered excellent. Meanwhile, the SRMR (0.08) indicated a moderate fit, as it is at the acceptable threshold of 0.08; values up to 0.09 are still considered acceptable in some contexts. Thus, results suggest that the proposed model provides a plausible framework for interpreting the relationships between latent variables and clinical outcomes.

The regressions from the structural model indicated that PRFs were significantly associated with all analyzed outcome variables (Table 3). The model showed that PRFs positively predicted the occurrence of musculoskeletal symptoms in the last 12 months (β = 0.40; p < 0.001), impairments due to these symptoms (β = 0.34; p = 0.001), musculoskeletal symptoms in the last 7 days (β = 0.32; p < 0.001), and medical visits in the last 12 months (β = 0.52; p < 0.001).

**Table 3:** Structural model regressions

Dependent variable	Estimate	SE	z-value	p-value	Std.lv	Std.all
Symptoms 12 months	1.04	0.24	4.20	0.000	1.01	0.40
Impairments 12 months	0.54	0.16	3.57	0.000	0.56	0.36
Medical care 12 months	1.18	0.21	5.52	0.000	1.14	0.52
Symptoms 7 days	0.66	0.15	4.18	0.000	0.64	0.32

SE = standard error; Std.all = standardized estimate (overall); Std.lv = standardized latent variable estimate.

The standardized coefficients indicated that the impact of PRFs was strongest on medical visits in the last 12 months (β = 0.52) and the occurrence of musculoskeletal symptoms in the last year (β = 0.40), followed by work impairments related to symptoms (β = 0.34) and the occurrence of symptoms in the last week (β = 0.32).

All z-values exceeded 3.25, confirming the statistical significance of these associations. These results suggest that higher levels of PRFs are associated with an increased frequency of musculoskeletal symptoms and their functional consequences, including work impairments and the need for medical care, reinforcing the importance of assessing psychosocial factors in the workplace.

## DISCUSSION

The results of this study corroborate the existing literature on the impact of PRFs on the health of workers.^12^,^13^ The high prevalence of musculoskeletal symptoms among participants (68.03%) aligns with previous research indicating that teaching is a profession with high physical and mental demands.^6^,^11^ The frequent occurrence of pain in the upper and lower back, as well as in the neck and shoulders, suggests the presence of postural overload and repetitive movements - common factors in academic work due to prolonged hours spent in front of a computer and the preparation of teaching materials.^28^

The significant association between PRFs and musculoskeletal symptoms supports that factors such as job insecurity, role overload, and work-family conflict not only affect professors’ emotional well-being but also their physical health.^4^,^10^,^29^ Job insecurity emerged as one of the strongest predictors of musculoskeletal symptoms (β = 0.40, p < 0.001), which may be related to the destabilization of employment relationships and the instability of the educational job market. This instability creates an environment characterized by constant pressure, directly impacting professors’ stress perception and, consequently, their musculoskeletal health.^8^,^9^

Work-family conflict also showed a strong relationship with musculoskeletal outcomes (β = 0.38, p < 0.001), suggesting that difficulties in balancing academic and personal responsibilities may increase stress levels. This finding is consistent with evidence that chronic stress leads to physiological changes, including increased muscle tension and a greater propensity for chronic pain.^4^,^7^,^10^,^13^ Lack of social support in the workplace also proved to be a relevant factor, particularly in relation to seeking medical care, reinforcing the hypothesis that less supportive work environments may hinder the adoption of healthy coping strategies.^4^,^15^

SEM results demonstrated that PRFs have a significant impact on musculoskeletal symptoms and their functional consequences, such as work absences and medical visits. These findings highlight the importance of effective management of psychosocial risks in HEIs, promoting interventions to reduce work overload and increase institutional support. Implementing policies that ensure greater job stability and offer emotional support to faculty staff members could be an effective strategy for reducing the incidence of musculoskeletal problems in this professional group.

## CONCLUSIONS

This study demonstrated that PRFs are significantly associated with the occurrence of musculoskeletal symptoms in university professors from the private sector, emphasizing the importance of managing these risks to promote occupational health. These findings align with the regulatory advancements represented by NR-1, which is an important step toward the management of psychosocial risks. However, more concrete institutional policies are still needed to mitigate these impacts.

The implications of these findings are relevant for both institutional policies and preventive measures, indicating the need to review working conditions in higher education. The adoption of psychological support programs, improvements in ergonomic conditions, and reductions in work overload are suggested as intervention strategies.

Among the limitations of this study, the cross-sectional nature of the research prevents definitive causal inferences. Additionally, the sample being restricted to a single institution may limit the generalizability of the results. Based on these limitations, future research should focus on longitudinal studies to clarify causal relationships between psychosocial factors and faculty musculoskeletal health, as well as investigate possible coping strategies to minimize the negative impacts of academic work.
